# Applying the termination of resuscitation rules to out-of-hospital cardiac arrests of both cardiac and non-cardiac etiologies: a prospective cohort study

**DOI:** 10.1186/s13054-016-1226-4

**Published:** 2016-03-01

**Authors:** Masahiro Kashiura, Yuichi Hamabe, Akiko Akashi, Atsushi Sakurai, Yoshio Tahara, Naohiro Yonemoto, Ken Nagao, Arino Yaguchi, Naoto Morimura, on behalf of the SOS-KANTO 2012 Study Group

**Affiliations:** Tertiary Emergency Medical Center, Tokyo Metropolitan Bokutoh Hospital, 4-23-15 Kotobashi, Sumida-ku, 130-8575 Tokyo Japan; Division of Emergency and Critical Care Medicine, Department of Acute Medicine, Nihon University School of Medicine, 30-1 Oyaguchikamicho, Itabashi-ku, 173-0032 Tokyo Japan; National Cerebral and Cardiovascular Center Hospital, 5-7-1 Fujishiro-dai, Suita, 565-8565 Osaka Japan; National Center of Neurology and Psychiatry, 4-1-1 Ogawa-Higashi, Kodaira, 187-8551 Tokyo Japan; Nihon University Surugadai Hospital, 1-6 Kanda-Surugadai, Chiyoda-ku, 101-8309 Tokyo Japan; Department of Critical Care and Emergency Medicine, Tokyo Women’s Medical University Hospital, 8-1 Kawadacho, Shinjuku-ku, 162-8666 Tokyo Japan; Department of Emergency Medicine, Yokohama City University Medical Center, 4-57 Urafunecho, Minami-ku, Yokohama-City, 232-0024 Kanagawa Japan

**Keywords:** Cardiopulmonary resuscitation, Decision support techniques, Emergency medical services, Out-of-hospital cardiac arrest

## Abstract

**Background:**

The 2015 American Heart Association Guidelines for Cardiopulmonary Resuscitation recommend Basic Life Support (BLS) and Advanced Life Support (ALS) rules for termination of resuscitation (TOR). However, it is unclear whether the TOR rules are valid for out-of-hospital cardiac arrests (OHCAs) of both cardiac and non-cardiac etiologies. In this study, we validated the TOR rules for OHCA resulting from both etiologies.

**Methods:**

This was a prospective multicenter observational study of OHCA patients transported to 67 emergency hospitals between January 2012 and March 2013 in the Kanto region of Japan. We calculated the specificity and positive predictive value (PPV) for neurologically unfavorable outcomes at one month in patients with OHCA of cardiac and non-cardiac etiologies.

**Results:**

Of 11,505 eligible cases, 6,138 and 5,367 cases were of cardiac and non-cardiac etiology, respectively. BLS was performed on 2,818 and 2,606 patients with OHCA of cardiac and non-cardiac etiology, respectively. ALS was performed on 3,320 and 2,761 patients with OHCA of cardiac and non-cardiac etiology, respectively. The diagnostic accuracy of the TOR rules for predicting unfavorable outcomes in patients with OHCA of cardiac etiology who received BLS included a specificity of 0.985 (95 % confidence interval [CI]: 0.956–0.997) and a PPV of 0.999 (95 % CI: 0.996–1.000). In patients with OHCA from cardiac etiologies who received ALS, the TOR rules had a specificity of 0.963 (95 % CI: 0.896–0.992) and a PPV of 0.997 (95 % CI: 0.991–0.999). In patients with OHCA from non-cardiac etiologies who received BLS, the specificity was 0.915 (95 % CI: 0.796–0.976) and PPV was 0.998 (95 % CI: 0.995–0.999). For patients with OHCA from non-cardiac etiologies who received ALS, the specificity was 0.833 (95 % CI: 0.586–0.964) and PPV was 0.996 (95 % CI: 0.988–0.999).

**Conclusions:**

Both TOR rules have high specificity and PPV in patients with OHCA from cardiac etiologies. For patients with OHCA from non-cardiac etiologies, the rules had a high PPV, but relatively low specificity. Therefore, TOR rules are useful in patients with OHCA from cardiac etiologies, but should be applied with caution to patients with OHCA from non-cardiac etiologies.

**Electronic supplementary material:**

The online version of this article (doi:10.1186/s13054-016-1226-4) contains supplementary material, which is available to authorized users.

## Background

Out-of-hospital cardiac arrest (OHCA) is a major public health problem worldwide. Approximately 330,000 individuals in the United States and 275,000 individuals in Europe experience OHCAs each year [1, 2]. The survival rate has steadily improved in OHCA cases from cardiac etiologies, whereas the survival rate for OHCA from all etiologies is about 10 %, similar to the rate three decades ago [1, 3, 4]. Most patients who survive an OHCA are resuscitated in the prehospital setting and are subsequently transported to emergency hospitals. Transporting all OHCA patients to emergency hospitals results in unnecessary consumption of valuable resources and exposes paramedics and the public to the risks of high-speed transportation [5].

Several studies have been conducted to prospectively determine and test the unequivocal termination of resuscitation (TOR) rules. The 2015 American Heart Association (AHA) Guidelines Update for Cardiopulmonary Resuscitation and Emergency Cardiovascular Care recommends that emergency medical service (EMS) personnel in prehospital settings should follow TOR rules in the protocols for basic life support (BLS) and advanced life support (ALS) [6]. The BLS TOR rule has three criteria, and all three of the following criteria must be present before terminating BLS resuscitative attempts for adult patients with OHCA: arrest was not witnessed by EMS personnel; no return of spontaneous circulation (ROSC) in the field; and no shock was delivered [7]. The ALS TOR rule recommends considering terminating resuscitation efforts when all of the following four criteria are met in the field: arrest was not witnessed; bystander cardiopulmonary resuscitation (CPR) was not provided; no ROSC after ALS care in the field; and no shock was delivered [8].

In a prospective study, the BLS TOR rule was 100 % predictive of death [9]. In several validation studies, both TOR rules were reported to have a high specificity and positive predictive value (PPV) for death or poor neurological outcomes [7, 10, 11]. The implementation of the TOR rules significantly reduces the rate of transport of futile OHCA. However, the European Resuscitation Council (ERC) guideline challenged the TOR rules and argued that applying the TOR rules led to an unexpected survival of 3.4–9 % of OHCA patients without sustained ROSC in the prehospital setting [12, 13]. Moreover, these validation studies were performed in patients with OHCA of presumed cardiac etiology and excluded patients with OHCA from noncardiac etiologies, such as suffocation, pulmonary embolism, incidental hypothermia, and vascular disease [7, 9–11]. Although the TOR rules have been validated in patients with OHCA from all etiologies in small population studies, there have been no studies specifically focused on OHCA of noncardiac etiology [14, 15]. As a result, it remains unclear whether the TOR rules are useful in patients with OHCA both of cardiac and noncardiac etiologies.

In this study, we assessed the validity of the BLS and ALS TOR rules for patients suffering from OHCA of cardiac or noncardiac etiology using data from the Survey of Survivors after Cardiac Arrest, which was collected in the Kanto Area of Japan in 2012 (SOS-KANTO 2012).

## Methods

### Setting and design

SOS-KANTO 2012 is a prospective, multicenter (including 67 emergency hospitals) observational study conducted between January 2012 and March 2013 in the Kanto region of Japan. The design and data collection methods used in SOS-KANTO 2012 have been reported in detail in prior studies [16, 17]. The Kanto region is made up of primarily urban areas, including Tokyo. The institutional ethics committees of each participating institution approved the study with a waiver for informed consent in order to protect participant anonymity.

### The EMS system in Japan

The EMS system in Japan has been explained in several previous studies [11, 18]. The EMS system is supervised by the Fire and Disaster Management Agency of Japan and is operated by each municipal government. All EMS personnel are certified to perform CPR according to the Japanese resuscitation guidelines produced by the Japan Resuscitation Council. Generally, the ambulance crew consists of three EMS personnel, including at least one emergency lifesaving technician (ELST). ELSTs may perform several resuscitation methods under the supervision of online medical control, including the operation of a semiautomated external defibrillator, insertion of a supraglottic airway device, and insertion of a peripheral intravenous line. Specially trained ELSTs have been able to perform endotracheal intubation since 2004 and to administer adrenaline intravenously under the supervision of online medical control since 2006. EMS personnel in Japan are not legally permitted to terminate resuscitation in the field, and all OHCA patients are transported to hospitals, except in cases where death is certain [18, 19].

### Participants

All OHCA patients transported by EMS personnel to participating institutions during the study were eligible for inclusion in SOS-KANTO 2012. Of these patients, the present study included only adults (18 years or older) suffering from nontraumatic OHCA. In addition, the following cases were excluded: cases with missing data regarding inclusion criteria or main outcomes (e.g., age, etiology of cardiac arrest, outcomes 1 month after cardiac arrest); cases with initial resuscitation performed inside the hospital and/or missing data for onset location; cases in which it was unknown whether BLS or ALS was performed by EMS personnel; and cases with missing data regarding BLS/ALS TOR criteria. Eligible participants were divided into two groups: those with OHCA from cardiac etiologies, and those with OHCA from noncardiac etiologies. Cases involving BLS and ALS were evaluated according to the BLS and ALS TOR rules, respectively.

### Data collection and definition

The EMS personnel collected prehospital data based on the Utstein-style template [20]. The absence of prehospital ROSC, one component of the TOR rule, was defined as no ROSC despite resuscitation effort of the EMS personnel until hospital arrival. Physicians collected data regarding in-hospital treatments and outcomes, and determined the etiology of OHCA, including cardiac (presumed cardiac origin) and noncardiac (e.g., asphyxia, trauma, aortic disease, drawing, cerebrovascular disease, and drug overdose). If more than one etiology was possible (e.g., ventricular fibrillation arrest leading to a fall from height), the most likely primary cause was recorded at the discretion of physicians.

### Outcome measurements

The primary outcome was survival with favorable neurological outcomes 1 month after cardiac arrest, defined as a Glasgow–Pittsburgh cerebral-performance category of 1 (good performance) or 2 (moderate disability) on a five-category scale [21, 22]. The other categories of 3 (severe disability), 4 (vegetative state), and 5 (death) were defined as unfavorable neurological outcomes. The secondary outcome was mortality 1 month after cardiac arrest.

### Statistical analysis

Descriptive statistics were calculated for all variables of interest. Continuous variables were reported as means and standard deviations, whereas categorical variables were summarized using counts and percentages. The BLS and ALS TOR rules were evaluated as diagnostic tests. The test characteristics, including sensitivity, specificity, false-positive rate (FPR), PPV and negative predictive value, were reported using 95 % confidence intervals (CIs). Lower sensitivity meant that more patients, who eventually died, were predicted to have good outcomes. AHA and ERC guidelines recommend the FPR to be close to 0 % and classify the reporting imprecision of a diagnostic test for prognostication as serious when the upper limit of the 95 % CI is >10 % [23, 24]. Statistical analysis was performed using IBM SPSS for Mac Version 22.0 (IBM Corp., Armonk, NY, USA).

## Results

**Fig. 1:**
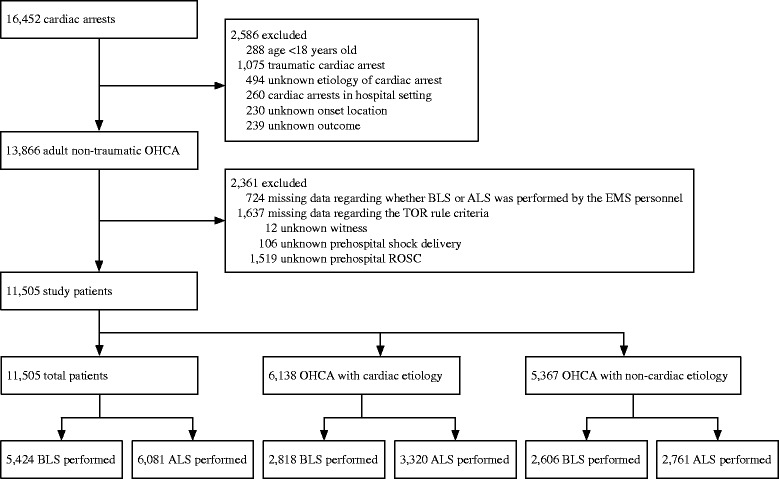
Flow diagram of the study population. The population for this study was obtained from data in the Survey of Survivors after Cardiac Arrest, which were collected in the Kanto Area of Japan from January 2012 to March 2013. *ALS* advanced life support, *BLS* basic life support, *EMS* emergency medical service, *OHCA* out-of-hospital cardiac arrest, *ROSC* return of spontaneous circulation, *TOR* termination of resuscitation

The study flow chart is shown in Fig. 1. During the survey period, a total of 16,452 patients with OHCA were documented. Of these, the following cases were excluded: 288 patients aged <18 years, 1075 patients with traumatic OHCA, 494 patients with missing etiology of arrest data, 239 cases with missing outcome data, 260 patients who initially received CPR in the hospital, and 230 cases with missing data for onset location. Therefore, 13,866 cases of nontraumatic adult OHCA were eligible for inclusion. However, an additional 724 cases were excluded because it was unknown whether BLS or ALS had been performed, and 1637 cases were excluded due to missing data regarding the TOR criteria (12 cases, unknown witnessed events; 106 cases, unknown shock delivery; and 1519 cases, unknown prehospital ROSC). As a result, 11,505 patients were enrolled in the present study. These patients were divided into two groups, with 6138 cases of OHCA from presumed cardiac etiologies and 5367 cases of OHCA from noncardiac etiologies. In total, 5424 patients received BLS (2818 cardiac cases and 2606 noncardiac cases) and 6081 patients received ALS (3320 cardiac cases and 2761 noncardiac cases).

The patient characteristics are presented in Table 1. The mean patient age was 72.4 years, and 6884 patients were men (59.8 %). Cardiac arrest occurred at home in 8395 cases (73.0 %). The mean time from call to ambulance arrival on the scene was 8.0 minutes, and the mean time from call to hospital arrival was 35.3 minutes. In 845 cases (7.3 %), the initial observed rhythm upon arrival was ventricular fibrillation or ventricular tachycardia. Advanced airways were placed in 5368 patients (46.7 %), and adrenaline was administered to 2287 patients (19.9 %). In total, BLS was performed on 5424 patients (47.1 %) and ALS was performed on 6081 patients (52.9 %).

**Table 1:** Clinical and prehospital characteristics of out-of-hospital cardiac arrest patients by group

Characteristic		Total (*n* = 11,505)	Cardiac etiology (*n* = 6138)	Noncardiac etiology (*n* = 5367)
Age (years)		72.4 ± 15.8	73.4 ± 14.5	71.2 ± 17.1
Male		6884 (59.8)	3827 (62.3)	3057 (57.0)
Location of arrest				
	Home	8395 (73.0)	4365 (71.1)	4030 (75.1)
	Public space (indoor)	352 (3.1)	217 (3.5)	135 (2.5)
	Public space (outdoor)	622 (5.4)	435 (7.1)	187 (3.5)
	Other	2136 (18.6)	1121 (18.3)	1015 (18.9)
EMS care interval (minutes)				
	Call to arrival of ambulance at scene	8.0 ± 3.6	8.0 ± 3.7	7.9 ± 3.6
	Call to hospital arrival	35.3 ± 3.6	35.4 ± 11.0	35.3 ± 12.0
Initial rhythm				
	Ventricular fibrillation/ventricular tachycardia	845 (7.3)	752 (12.3)	93 (1.7)
	Pulseless electrical activity	2369 (20.6)	1111 (18.1)	1258 (23.4)
	Asystole	7550 (65.6)	3909 (63.7)	3641 (67.8)
	Others or unknown	741 (6.4)	366 (6.0)	375 (7.0)
Advanced airway procedure		5368 (46.7)	2941 (47.9)	2427 (45.2)
Prehospital adrenaline administration		2287 (19.9)	1220 (19.9)	1067 (19.9)
BLS performed by EMS		5424 (47.1)	2818 (46.9)	2606 (48.6)
	Met BLS TOR criteria	4193	2060	2133
ALS performed by EMS		6081 (52.9)	3320 (54.1)	2761 (51.4)
	Met ALS TOR criteria	1701	956	745
Arrest witnessed by				
	Bystander	5473 (47.6)	2881 (46.9)	2592 (48.3)
	EMS/first responder	929 (8.1)	480 (7.8)	449 (8.4)
Prehospital defibrillation (layperson and EMS)		1565 (13.6)	1249 (20.3)	316 (5.9)
Bystander-initiated CPR		4231 (36.8)	2226 (36.3)	2005 (37.4)
Prehospital ROSC		1070 (9.3)	570 (9.3)	500 (9.3)

Data presented as means ± standard deviations for continuous variables and numbers (percentage) for categorical values

*ALS* advanced life support, *BLS* basic life support, *CPR* cardiopulmonary resuscitation, *EMS* emergency medical service, *ROSC* return of spontaneous circulation, *TOR* termination of resuscitation

Regarding the TOR rules criteria, cardiac arrests were witnessed by bystanders and EMS personnel in 5473 (47.6 %) cases and 929 (8.1 %) cases, respectively. Prehospital defibrillation was delivered in 1565 cases (13.6 %), bystander-initiated CPR was performed in 4231 cases (36.8 %), and ROSC was achieved in the prehospital setting in 1070 cases (9.3 %). In summary, 4193 and 1701 patients met all BLS and ALS TOR criteria, respectively.

Characteristics regarding in-hospital treatment and outcome data are presented in Table 2. Shocks were delivered in the emergency room in 1144 cases (9.9 %), and adrenaline was administered in 9086 cases (79.0 %). Of the 2321 patients (20.2 %) who survived until hospital admission, coronary angiography and therapeutic hypothermia was performed in 520 cases (22.4 %) and 606 cases (26.1 %), respectively. One month after admission, 11,161 cases (97.0 %) had unfavorable neurological outcomes, and 10,933 patients (95.0 %) had died.

**Table 2:** In-hospital management and 1-month outcomes for out-of-hospital cardiac arrest patients by group

Characteristic		Total (*n* = 11,505)	Cardiac etiology (*n* = 6138)	Noncardiac etiology (*n* = 5367)
Defibrillation in ER		1144 (9.9)	831 (13.5)	313 (5.8)
Adrenaline administration in ER		9086 (79.0)	4819 (78.5)	4267 (79.5)
Survival until hospital admission		2321 (20.2)	1112 (18.1)	1209 (22.5)
Coronary angiography^a^		520 (22.4)	483 (43.4)	37 (3.1)
Therapeutic hypothermia^a^		606 (26.1)	437 (39.3)	169 (14.0)
Clinical outcomes 1 month after admission				
	Neurologically favorable outcome (CPC 1 or 2)	344 (3.0)	279 (4.5)	65 (1.2)
	Neurologically unfavorable outcome (CPC 3–5)	11,161 (97.0)	5859 (95.5)	5302 (98.8)
	Survival	572 (5.0)	410 (6.7)	162 (3.0)
	Death	10,933 (95.0)	5728 (93.3)	5205 (97.0)

Data presented as numbers (percentage) for categorical values

^a^Proportion calculated using the numbers of patients who survived until hospital admission as the denominator

*CPC* cerebral performance category, *ER* emergency room

### Diagnostic accuracy of the TOR rules for all patients

The characteristics of the diagnostic tests for the TOR rules for 1-month neurological outcomes and mortality are presented in Tables 3 and 4, respectively. For all cases (OHCA from both cardiac and noncardiac etiologies), the BLS TOR rule had a specificity of 0.971 (95 % CI: 0.942–0.988), a FPR of 2.9 % (95 % CI: 1.2–5.8 %), and a PPV of 0.998 (95 % CI: 0.997–0.999) for predicting neurologically unfavorable outcomes at 1 month (Table 3). In this same group, the BLS TOR rules had a specificity of 0.918 (95 % CI: 0.884–0.945), a FPR of 8.2 % (95 % CI: 5.5–11.6 %), and a PPV of 0.993 (95 % CI: 0.990–0.996) for 1-month mortality (Table 4). The ALS TOR rule in patients with OHCA from both cardiac and noncardiac etiologies had a specificity of 0.939 (95 % CI: 0.873–0.977), a FPR of 6.1 % (95 % CI: 2.3–12.7 %), and a PPV of 0.996 (95 % CI: 0.992–0.999) for predicting neurologically unfavorable outcomes at 1 month (Table 3). The ALS TOR rule had a specificity of 0.913 (95 % CI: 0.869–0.946), a FPR of 8.7 % (95 % CI: 5.4–13.1 %), and a PPV of 0.988 (95 % CI: 0.982–0.993) for 1-month mortality (Table 4).

**Table 3:** Diagnostic accuracy of termination of resuscitation rules for 1-month neurological outcomes

Etiology of cardiac arrest		Neurologically unfavorable outcome	Neurologically favorable outcome	Sensitivity (95 % CI)	Specificity (95 % CI)	FPR (95 % CI)	PPV (95 % CI)	NPV (95 % CI)
BLS TOR rule								
All types								
	Met criteria	4186	7	0.808	0.971	2.9 %	0.998	0.193
	Did not meet criteria	993	238	(0.797–0.819)	(0.942–0.988)	(1.2–5.8 %)	(0.997–0.999)	(0.172–0.217)
Cardiac etiology								
	Met criteria	2057	3	0.785	0.985	1.5 %	0.999	0.257
	Did not meet criteria	563	195	(0.769–0.801)	(0.956–0.997)	(0.3–4.4 %)	(0.996–1.000)	(0.226–0.290)
Noncardiac etiology								
	Met criteria	2129	4	0.832	0.915	8.5 %	0.998	0.091
	Did not meet criteria	430	43	(0.817–0.846)	(0.796–0.976)	(2.4–20.4 %)	(0.995–0.999)	(0.067–0.121)
ALS TOR rule								
All types								
	Met criteria	1695	6	0.353	0.939	6.1 %	0.996	0.021
	Did not meet criteria	4287	93	(0.341–0.365)	(0.873–0.977)	(2.3–12.7 %)	(0.992–0.999)	(0.017–0.026)
Cardiac etiology								
	Met criteria	953	3	0.294	0.963	3.7 %	0.997	0.033
	Did not meet criteria	2286	78	(0.279–0.310)	(0.896–0.992)	(0.8–10.4 %)	(0.991–0.999)	(0.026–0.041)
Noncardiac etiology								
	Met criteria	742	3	0.271	0.833	16.7 %	0.996	0.007
	Did not meet criteria	2001	15	(0.254–0.288)	(0.586–0.964)	(3.6–41.4 %)	(0.988–0.999)	(0.004–0.012)

*ALS* advanced life support, *BLS* basic life support, *CI* confidence interval, *FPR* false-positive rate, *NPV* negative predictive value, *PPV* positive predictive value, *TOR* termination of resuscitation

**Table 4:** Diagnostic accuracy of termination of resuscitation rules for 1-month mortality

Etiology of cardiac arrest		Deaths	Survival	Sensitivity (95 % CI)	Specificity (95 % CI)	FPR (95 % CI)	PPV (95 % CI)	NPV (95 % CI)
BLS TOR rule								
All types								
	Met criteria	4165	28	0.820	0.918	8.2 %	0.993	0.255
	Did not meet criteria	917	314	(0.809–0.830)	(0.884–0.945)	(5.5–11.6 %)	(0.990–0.996)	(0.231–0.280)
Cardiac etiology								
	Met criteria	2044	16	0.798	0.938	6.2 %	0.992	0.318
	Did not meet criteria	517	241	(0.782–0.814)	(0.901–0.964)	(3.6–9.9 %)	(0.987–0.996)	(0.285–0.352)
Noncardiac etiology								
	Met criteria	2121	12	0.841	0.859	14.1 %	0.994	0.154
	Did not meet criteria	400	73	(0.826–0.855)	(0.766–0.925)	(7.5–23.4 %)	(0.990–0.997)	(0.123–0.190)
ALS TOR rule								
All types								
	Met criteria	1681	20	0.287	0.913	8.7 %	0.988	0.048
	Did not meet criteria	4170	210	(0.276–0.299)	(0.869–0.946)	(5.4–13.1 %)	(0.982–0.993)	(0.042–0.055)
Cardiac etiology								
	Met criteria	947	9	0.299	0.941	5.9 %	0.991	0.061
	Did not meet criteria	2220	144	(0.283–0.315)	(0.891–0.973)	(2.7–10.9 %)	(0.982–0.996)	(0.052–0.071)
Noncardiac etiology								
	Met criteria	734	11	0.273	0.857	14.3 %	0.985	0.033
	Did not meet criteria	1950	66	(0.257–0.291)	(0.759–0.926)	(7.4–24.1 %)	(0.974–0.993)	(0.025–0.042)

*ALS* advanced life support, *BLS* basic life support, *CI* confidence interval, *FPR* false-positive rate, *NPV* negative predictive value, *PPV* positive predictive value, *TOR* termination of resuscitation

### Diagnostic accuracy of the TOR rules for patients with OHCA of cardiac etiology

In patients with OHCA from cardiac etiologies, the BLS TOR rule had a specificity of 0.985 (95 % CI: 0.956–0.997), a FPR of 1.5 % (95 % CI: 0.3–4.4 %), and a PPV of 0.999 (95 % CI: 0.996–1.000) for predicting neurologically unfavorable outcomes at 1 month (Table 3). In this same group, the BLS TOR rules had a specificity of 0.938 (95 % CI: 0.901–0.964), a FPR of 6.2 % (95 % CI: 3.6–9.9 %), and a PPV of 0.992 (95 % CI: 0.987–0.996) for 1-month mortality (Table 4). The ALS TOR rule for patients with OHCA from cardiac etiologies had a specificity of 0.963 (95 % CI: 0.896–0.992), a FPR of 3.7 % (95 % CI: 0.8–10.4 %), and a PPV of 0.997 (95 % CI: 0.991–0.999) for predicting neurologically unfavorable outcomes at 1 month (Table 3). For 1-month mortality, the ALS TOR rule had a specificity of 0.941 (95 % CI: 0.891–0.973), a FPR of 5.9 % (95 % CI: 2.7–10.9 %), and a PPV of 0.991 (95 % CI: 0.982–0.996) (Table 4).

### Diagnostic accuracy of the TOR rules for patients with OHCA of noncardiac etiology

In patients with OHCA from noncardiac etiologies, the BLS TOR rule had a specificity of 0.915 (95 % CI: 0.796–0.976), a FPR of 8.5 % (95 % CI: 2.4–20.4 %), and a PPV of 0.998 (95 % CI: 0.995–0.999) for predicting neurologically unfavorable outcomes at 1 month (Table 3). In this same group, the BLS TOR rules had a specificity of 0.859 (95 % CI: 0.766–0.925), a FPR of 14.1 % (95 % CI: 7.5–23.4 %), and a PPV of 0.994 (95 % CI: 0.990–0.997) for mortality at 1 month (Table 4). The ALS TOR rule for patients with OHCA from noncardiac etiologies had a specificity of 0.833 (95 % CI: 0.586–0.964), a FPR of 16.7 % (95 % CI: 3.6–41.4 %), and a PPV of 0.996 (95 % CI: 0.988–0.999) for predicting neurologically unfavorable outcomes at 1 month (Table 3). For 1-month mortality, the ALS TOR rule had a specificity of 0.857 (95 % CI: 0.759–0.926), a FPR of 14.3 % (95 % CI: 7.4–24.1 %), and a PPV of 0.985 (95 % CI: 0.974–0.993) (Table 4).

### Characteristics of unexpected survivors with neurologically favorable outcome

Seven and six unexpected survivors had good neurological outcomes in the BLS and ALS TOR rules, respectively (see Additional file 1: Table S1). Of these 13 cases, incidental hypothermia, pulmonary embolism, drug overdose, and suffocation accounted for seven cases with noncardiac etiologies. Extracorporeal cardiopulmonary resuscitation (ECPR) was performed in five cases.

## Discussion

We investigated the diagnostic accuracy of the BLS and ALS TOR rules for predicting unfavorable neurological outcomes and mortality 1 month after OHCA using data from SOS-KANTO 2012. Both TOR rules showed a high specificity and PPV and a low FPR in patients with OHCA of cardiac etiology. However, the TOR rules had a high PPV but low specificity and high FPR in patients with OHCA of noncardiac etiology. The imprecision of the BLS and ALS TOR rules is summarized in Table 5.

**Table 5:** Imprecision of basic and advanced life support termination of resuscitation rules

	Etiology	Imprecision
For predicting 1-month unfavorable neurological outcome		
BLS TOR rule	Cardiac	Acceptable
	Noncardiac	Serious
ALS TOR rule	Cardiac	Serious
	Noncardiac	Serious
For predicting 1-month mortality		
BLS TOR rule	Cardiac	Acceptable
	Noncardiac	Serious
ALS TOR rule	Cardiac	Serious
	Noncardiac	Serious

Imprecision is classified as acceptable and serious when the upper limit of the 95 % confidence interval of the false-positive rate is ≤10 % and >10 %, respectively

*ALS* advanced life support, *BLS* basic life support, *TOR* termination of resuscitation

For patients in the cardiac etiology group, the diagnostic accuracy of the TOR rules was similar to results reported in prior studies conducted in North America and Japan [7, 9–11]. Similarly, the effectiveness of both TOR rules for predicting neurologically unfavorable outcomes and death after OHCA in Japan was demonstrated in this present study. In cases of OHCA due to cardiac etiology, both TOR rules were useful in determining when to terminate resuscitation efforts in the prehospital setting.

This study had several strengths, particularly regarding the population and sample size. Firstly, the efficacy of the TOR rules was validated in all patients transported to emergency hospitals in a densely populated area of Japan. Secondly, we included almost all OHCA patients in this area of Japan because EMS personnel in Japan are legally obligated to transport OHCA patients to the hospital, except in cases of obvious mortality [19, 25]. In previous studies of this topic, TOR occurred in approximately 17 % of patients in the prehospital setting [10].

For patients in the noncardiac etiology group, the specificity of both TOR rules for neurologically unfavorable outcomes and mortality at 1 month were relatively low. Conversely, the PPVs of both TOR rules for patients in the noncardiac etiology group were very high and similar to the PPVs for patients in the cardiac etiology group. The pretest probability for 1-month mortality and unfavorable outcomes was 97.0 % and 98.8 %, respectively; therefore a high PPV depends on a high pretest probability.

The etiologies of cardiac arrest were categorized clinically according to the Utstein-style guidelines for cardiac arrest data reporting [20]. Although the survival rate for patients with OHCA of noncardiac etiology is very poor, survival rates differ extensively for patients with OHCA of noncardiac etiology [26]. For instance, in patients with OHCA resulting from respiratory disease, asphyxia, drug overdose, and incidental hypothermia, the survival rate is higher than in those from other noncardiac etiologies [26]. In the present study, special circumstances, such as incidental hypothermia or drug overdose, were relatively common etiologies of noncardiac unexpected survivors. The ERC guideline also recommends that EMS personnel should consider continuing CPR during transport to hospital in patients with OHCAs with presumed reversible causes, such as drug overdose and hypothermia [12]. Moreover, it is often difficult to determine the etiology of cardiac arrest in the prehospital setting [27]. Cost-effectiveness is very important in the TOR rule. Previous researchers have reported that applying the TOR rules reduces approximately 40–50 % of futile transportation and further resuscitation efforts [7, 9]. However, the decision of the TOR is more challenging in contrast to other interventions. It has been argued that success rates of <1 % still justify the resuscitation effort [12]. According to our results, the TOR rules are less useful for OHCAs of noncardiac etiology than those of cardiac etiology. Until these discrepancies are resolved, it would be necessary to develop new TOR rules that are effective for OHCAs from both cardiac and noncardiac etiologies. At the least, we consider that the TOR rules should not be applied in cases of presumed reversible etiologies.

According to our results, the upper limit of the FPR of the ALS TOR rule was near 10 %, which is high for cardiac etiology. As advanced therapies (i.e., ECPR) become widely available, TOR rules require integration with guidance on suitability for these therapies [10, 28, 29]. For example, the low end-tidal carbon dioxide (EtCO_2_) value during CPR may indicate a poor prognosis [30–32]. An EtCO_2_ < 10 mmHg after 20 minutes of CPR may be considered a component of a multimodal approach to TOR, particular for withholding ECPR [33, 34]. Although EtCO_2_ was not evaluated in the present study, several unexpected survivors underwent ECPR. Therefore, consideration of EtCO_2_ may improve the ALS TOR rule.

This study had several limitations. Firstly, this was an observational study, not an interventional study. Although prehospital TOR is not legally permitted in Japan, resuscitation efforts might not be conducted consistently in all OHCA patients. Secondly, SOS-KANTO 2012 was not a population-based study because it was conducted only in the Kanto area of Japan. Therefore, it is possible that our findings are not generalizable to other parts of Japan or other countries. However, the characteristics and outcomes of OHCA patients in this study were similar to those in other population-based studies in Japan and other countries [7, 10, 11, 18]. Finally, this study excluded pediatric patients and those with traumatic cardiac arrest; therefore, the effectiveness of the prehospital TOR rules for these populations is unclear.

## Conclusions

The BLS and ALS TOR rules were useful for predicting neurologically unfavorable outcomes and mortality 1 month after the onset of OHCA of cardiac etiology. However, these rules should be applied cautiously to patients with OHCA of noncardiac etiology.

## Key messages

- The BLS and ALS TOR rules were useful for predicting unfavorable neurological outcomes 1 month after onset of OHCA of cardiac etiology, demonstrating both a high specificity and PPV.
- The TOR rules should be applied with caution to patients with OHCA of noncardiac etiologies because these rules were observed to have a high PPV but a relatively low specificity.

## Additional file

Additional file 1: Table S1. Presenting the characteristics of 13 unexpected survivors with favorable neurological outcomes. (DOCX 16 kb)

## Abbreviations

AHA: American Heart Association
ALS: Advanced life support
BLS: Basic life support
CI: Confidence interval
CPR: Cardiopulmonary resuscitation
ECPR: Extracorporeal cardiopulmonary resuscitation
ELST: Emergency lifesaving technician
EMS: Emergency medical service
ERC: European Resuscitation Council
EtCO_2_: End-tidal carbon dioxide
FPR: False-positive rate
OHCA: Out-of-hospital cardiac arrest
PPV: Positive predictive value
ROSC: Return of spontaneous circulation
SOS-KANTO 2012: Survey of Survivors after Cardiac Arrest in the Kanto Area in 2012
TOR: Termination of resuscitation
