# Supplementary material for: Applying the termination of resuscitation rules to out-of-hospital cardiac arrests of both cardiac and non-cardiac etiologies: a prospective cohort study
Source: Crit Care. 2016 Mar 1;20:49. doi: 10.1186/s13054-016-1226-4 (PMC4772485; doi:10.1186/s13054-016-1226-4)
Supplement: Additional file 1: Table S1. — Presenting the characteristics of 13 unexpected survivors with favorable neurological outcomes. (DOCX 16 kb) [file 13054_2016_1226_MOESM1_ESM.docx]

**Table S1:** The characteristics of 13 unexpected survivors with favorable neurological outcomes

| Case | EMS performed | Age, y | Sex | Etiology | Witness | Bystander-initiated CPR | Initial rhythm | ECPR | Coronary angiography | Therapeutic hypothermia |
| --- | --- | --- | --- | --- | --- | --- | --- | --- | --- | --- |
| 1 | BLS | 38 | Female | Cardiac | + | - | PEA | + | + | + |
| 2 | BLS | 64 | Male | Cardiac | + | + | PEA | + | + | - |
| 3 | BLS | 70 | Male | Cardiac | + | - | Asytole | - | + | + |
| 4 | ALS | 59 | Male | Cardiac | - | - | PEA | - | + | - |
| 5 | ALS | 52 | Male | Cardiac | - | - | PEA | - | - | - |
| 6 | ALS | 41 | Male | Cardiac | - | - | PEA | - | + | + |
| 7 | BLS | 66 | Male | Drug overdose | + | - | PEA | - | - | - |
| 8 | ALS | 83 | Male | Drug overdose | - | - | Asystole | - | - | - |
| 9 | ALS | 47 | Female | Incidental hypothermia | - | - | Asystole | + | - | + |
| 10 | ALS | 64 | Male | Incidental hypothermia | - | - | Asystole | - | + | + |
| 11 | BLS | 52 | Female | Pulmonary embolism | - | - | Asystole | + | - | - |
| 12 | BLS | 92 | Female | Pulmonary embolism | + | - | PEA | + | - | - |
| 13 | BLS | 46 | Male | Suffocation | + | - | PEA | - | - | + |

EMS, emergency medical service; BLS, basic life support; ALS, advanced life support; CPR, cardiopulmonary circulation; PEA, pulseless electrical activity; ECPR, extracorporeal cardiopulmonary resuscitation.
